# Supplementary material for: Structural basis for Ragulator functioning as a scaffold in membrane-anchoring of Rag GTPases and mTORC1
Source: Nat Commun. 2017 Nov 9;8:1394. doi: 10.1038/s41467-017-01567-4 (PMC5680233; doi:10.1038/s41467-017-01567-4)
Supplement: Supplementary file 1 — Supplementary Information [file 41467_2017_1567_MOESM1_ESM.pdf]

a

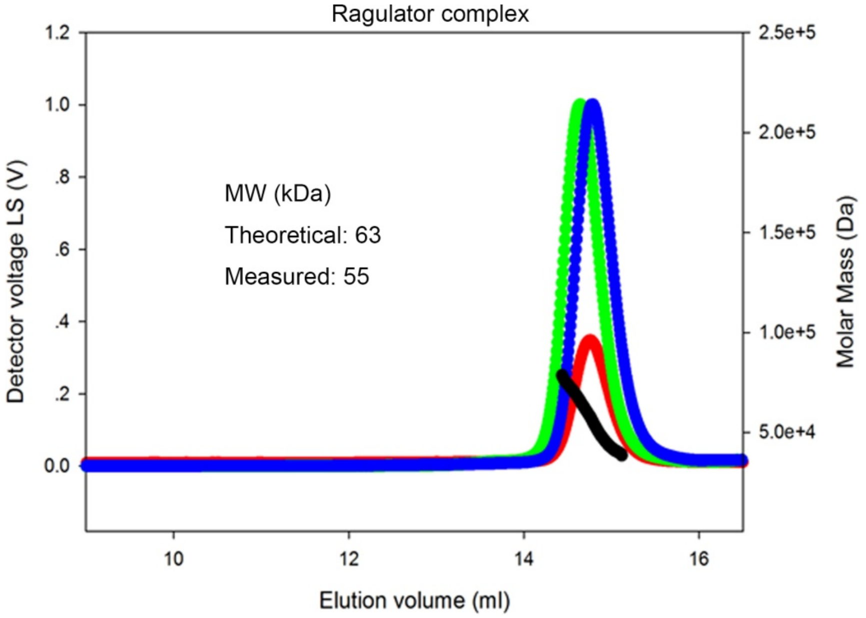

b

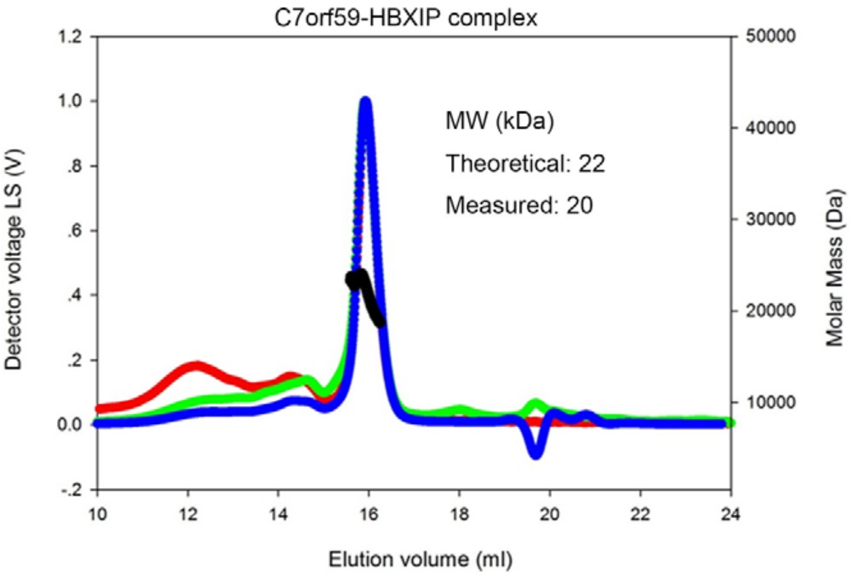

Supplementary Figure 1. Size-exclusion chromatography coupled with multi-angle light scattering (SEC-MALS) analysis of the Ragulator complex (p18$^{14\text{-}161}$) (**a**) and the C7orf59-HBXIP heterodimer (**b**). The left and right vertical axes represent the refractive index reading and the molecular mass. Chromatograms show the readings from the light scattering at 90° (red), refractive index (blue), and UV (green) detectors. The black curves represent the calculated

molecular masses, and the average measured masses of the elution peaks of the Ragulator complex ($p18^{14-161}$) and the C7orf59-HBXIP heterodimer are indicated, suggesting that both complexes exist as monomers in solution.

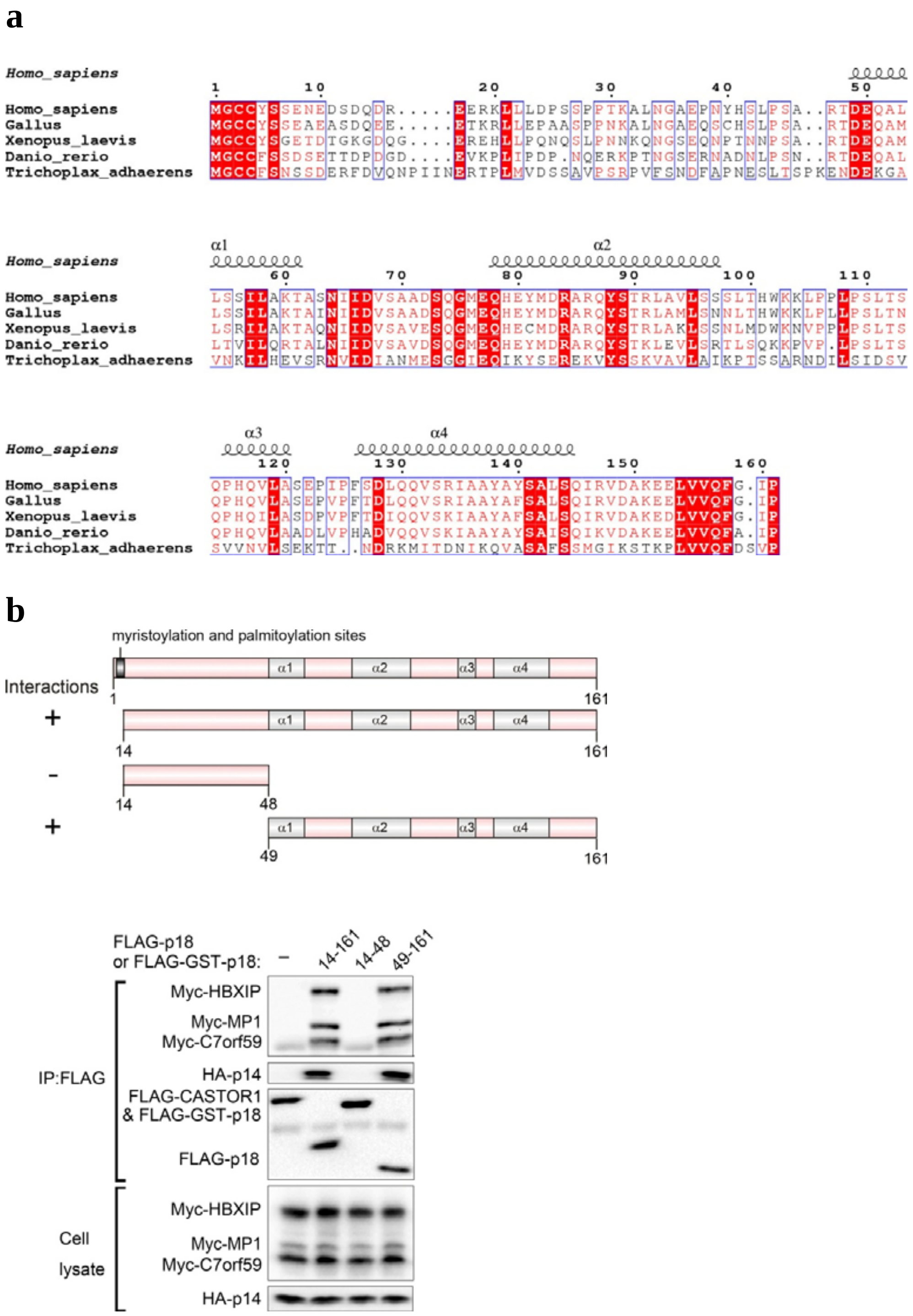

Supplementary Figure 2. Biochemical analysis of the interactions of different p18 variants with MP1/p14/HBXIP/C7orf59. (**a**) Sequence alignment of p18 from different species as analyzed by

ESPript 3.0[1]. The secondary structures (α2-α4) of p18 in the Ragulator complex are placed on the top of the alignment. Helix α1 was predicted by the PredictProtein server[2]. (**b**) Co-IP assays examining the interaction of the p18 delection mutants with the other four Ragulator components. The N-terminal trunaction of p18 ($p18^{49-161}$) exhibits similar binding affinity with the other components as $p18^{14-161}$.

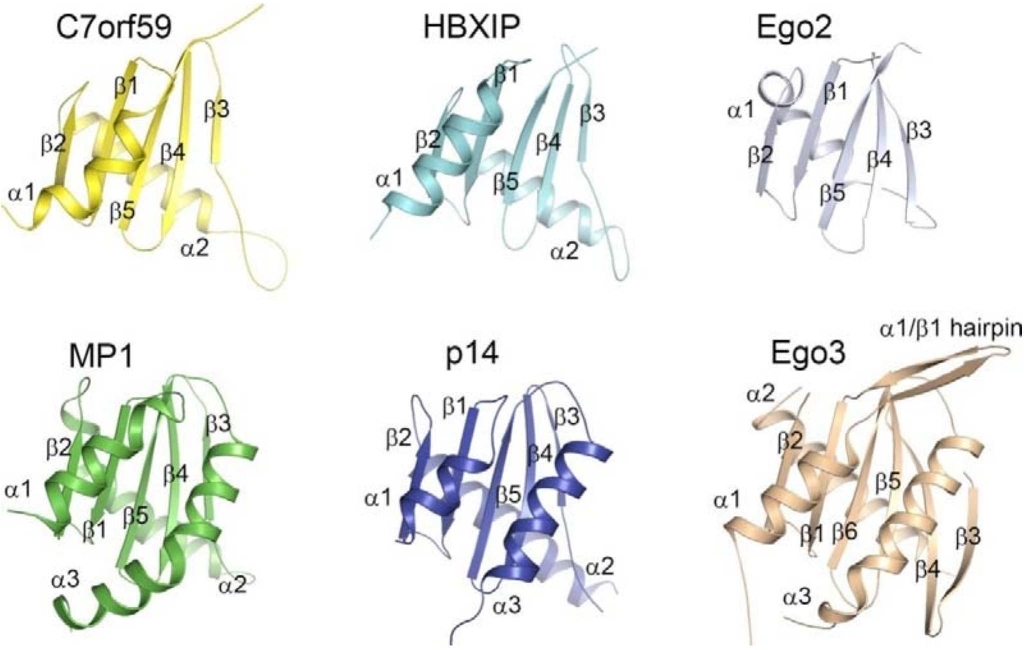

Supplementary Figure 3. Structures of the Roadblock domain-containing components in the Ragulator and EGO-TC complexes.

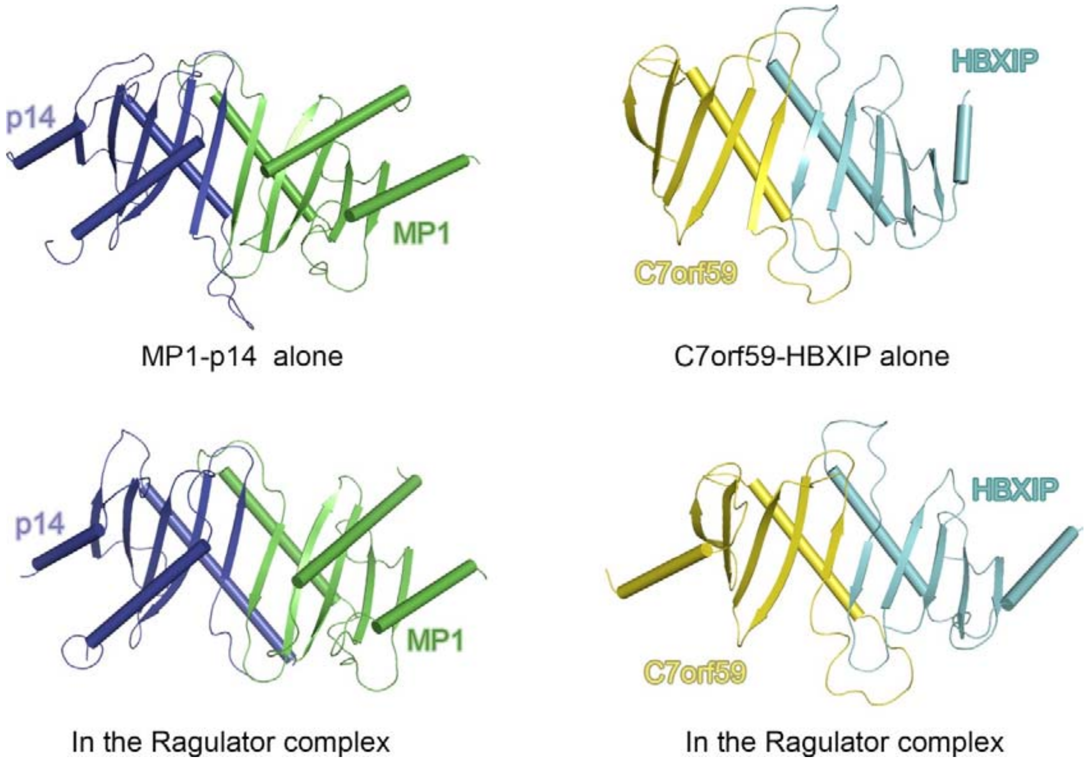

Supplementary Figure 4. Structural comparision of the C7orf59-HBXIP and MP1-p14 heterodimers alone and those in the Ragulator complex. The structure of Mp1-p14 (PDB code 1VET) is reported previously[3]. The color scheme is the same as in Figure 1.

a

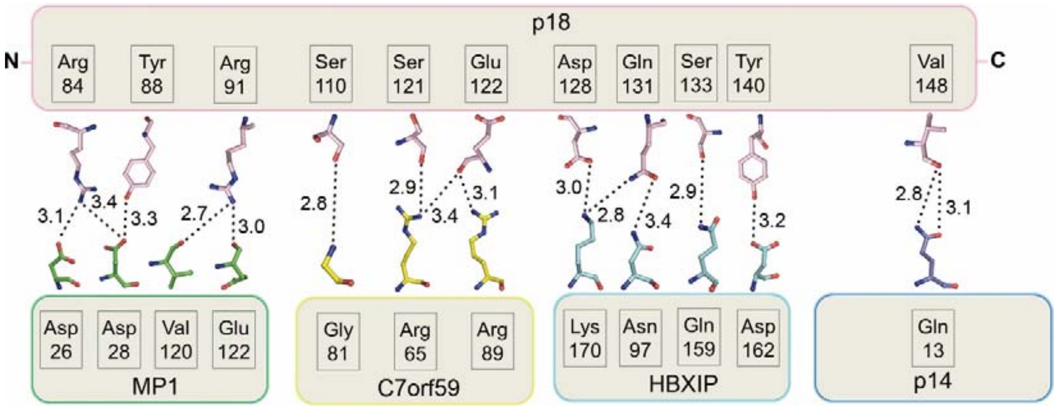

b

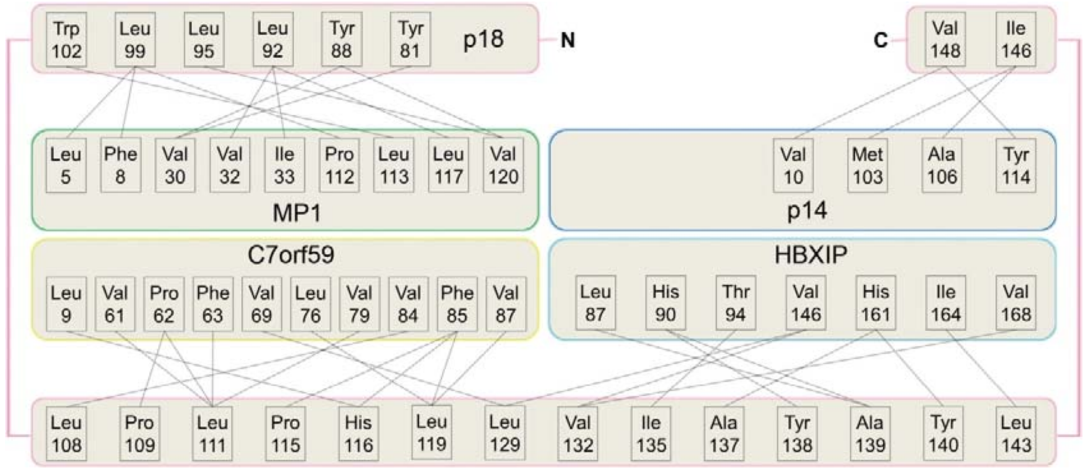

Supplementary Figure 5. Interactions of p18 with MP1/p14/HBXIP/C7orf59. Schematic diagrams showing the hydrophilic interactions (**a**) and hydrophobic interactions (**b**). The color scheme is the same as in Figure 2a.

a

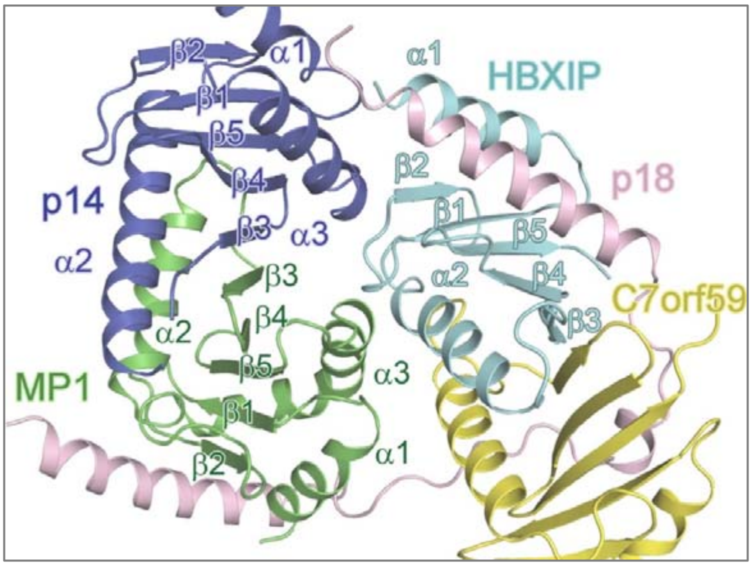

b

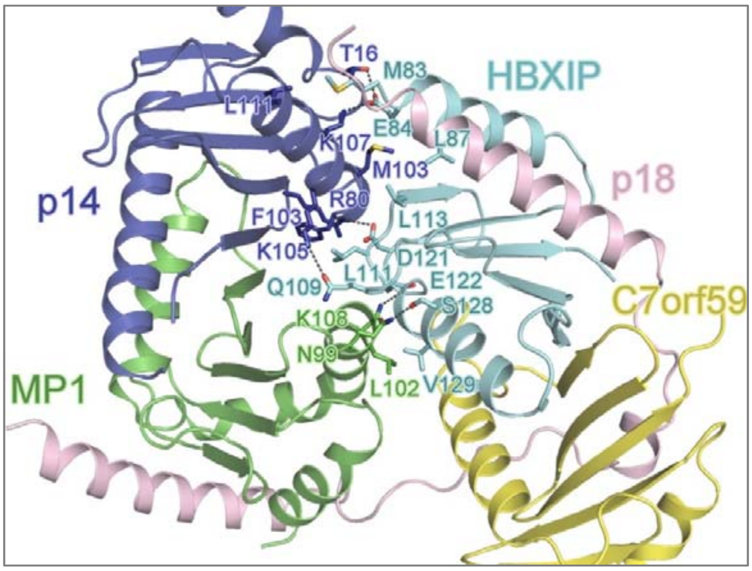

Supplementary Figure 6. Interactions between the MP1-p14 and HBXIP-C7orf59 heterodimers. Overview (**a**) and detailed view (**b**) of the interactions between the two heterodimers. The interaction interface is mediated by the structure elements of MP1, p14 and HBXIP but not C7orf59.

a

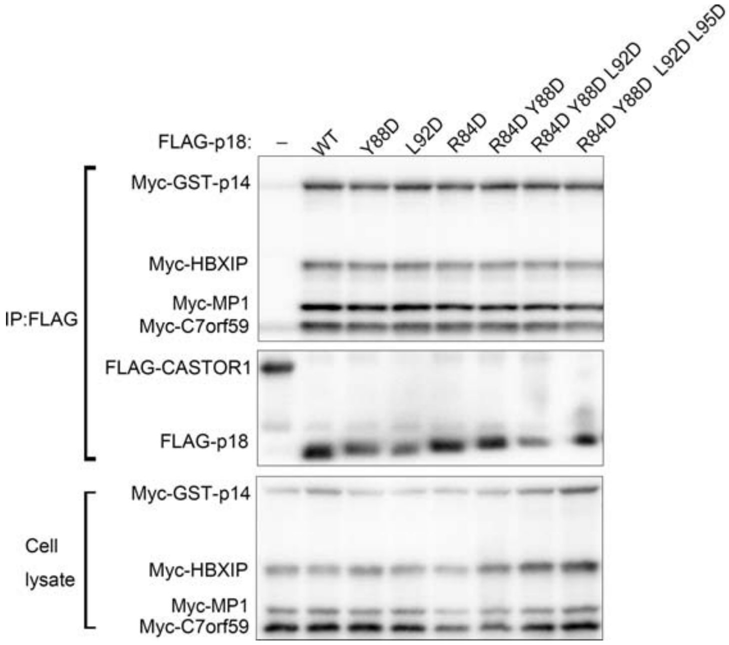

b

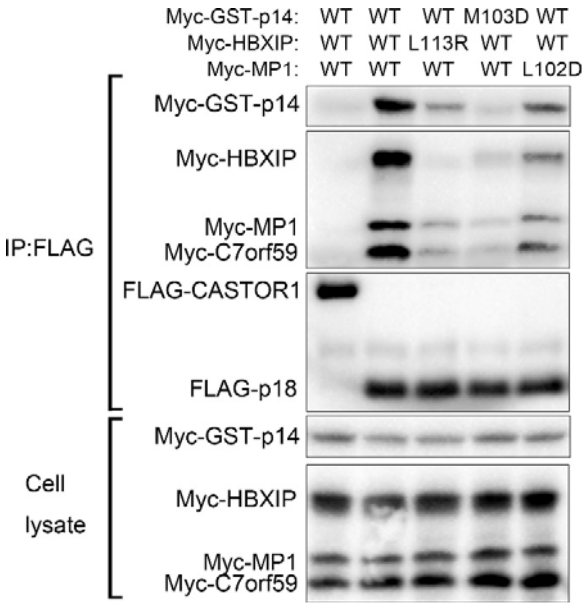

Supplementary Figure 7. Mutational analyses of the interactions among different Ragulator components. (**a**) Co-IP assays examining the interactions between p18 and MP1. All the mutations on the MP1-binding region of p18 do not impair the interactions. (**b**) Co-IP assays

examining the interactions between the MP1-p14 and C7orf59-HBXIP heterodimers. All the mutations on p14, HBXIP or MP1 weaken the interactions.

a

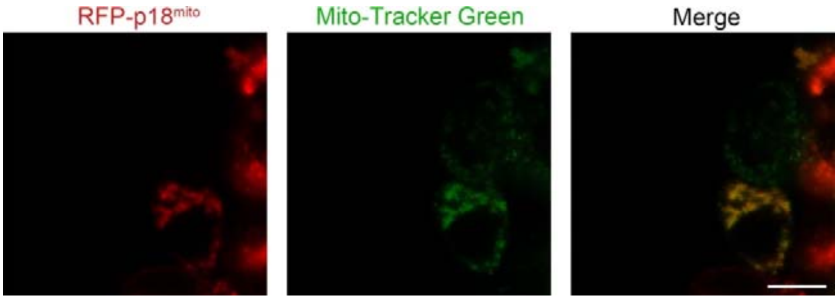

b

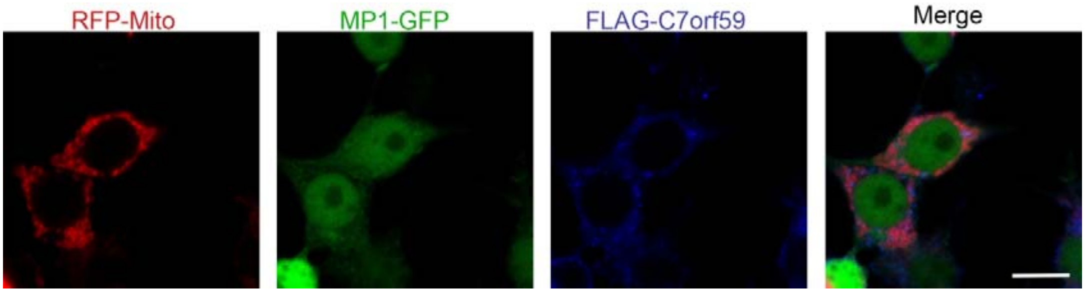

Supplementary Figure 8. Immunofluorescence microscopy analysis to examine the localization of p18^{mito}. p18^{mito} was constructed with the deletion of the N-terminal lipidation region (residues 1-13) and the fusion of the mitochondrial transmembrane region of OMP25 at the C-terminus. Scale bar represents 10 μm. (**a**) p18^{mito} was co-localized with the mitochondrial marker Mito-tracker Green. (**b**) RFP-Mito failed to recruit MP1 and C7orf59 in cells over-expressing MP1, p14, C7orf59 and HBXIP.

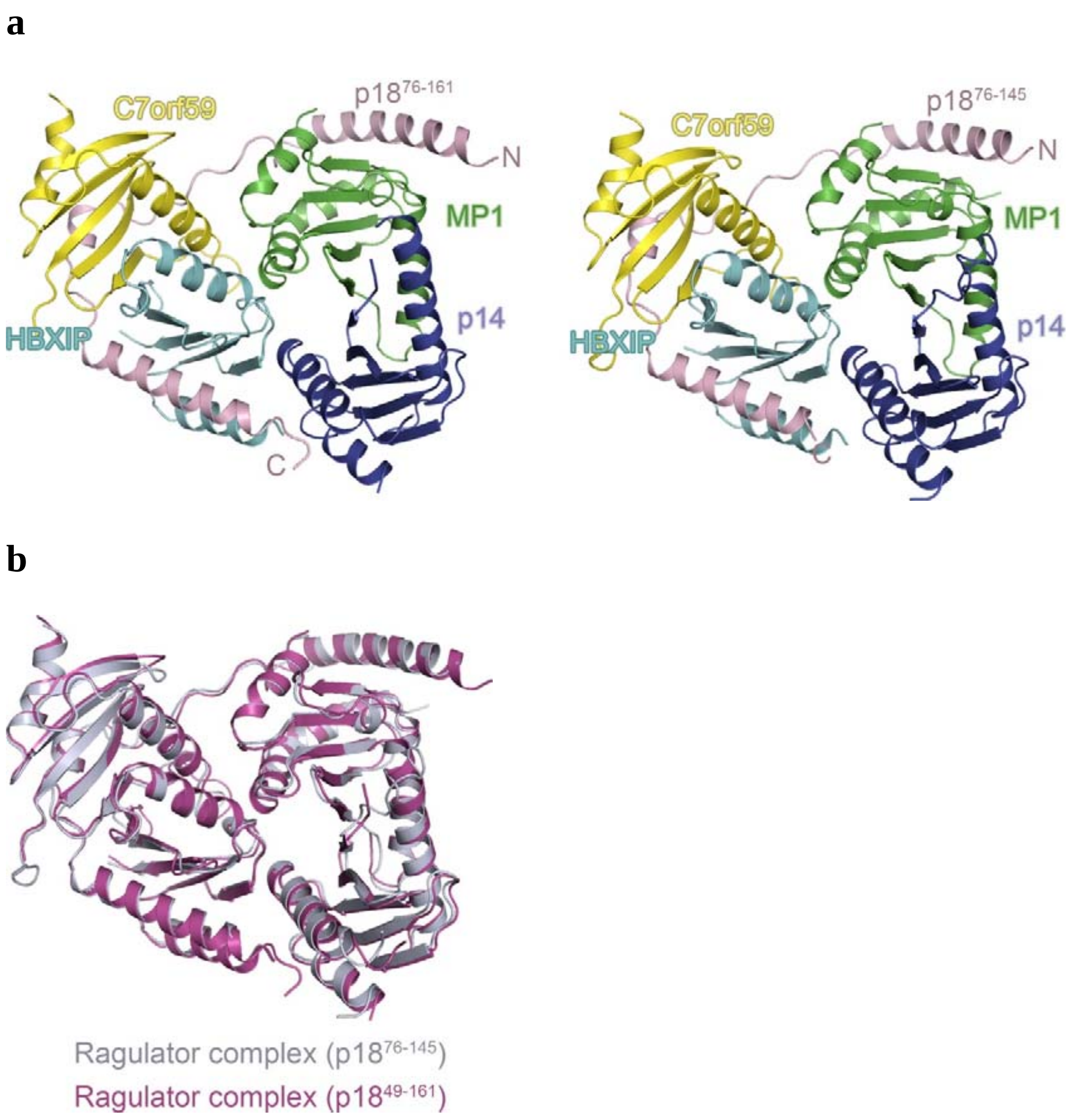

Supplementary Figure 9. Structural comparision of the Ragulator ($p18^{49\text{-}161}$) and Ragulator ($p18^{76\text{-}145}$) complexes. (**a**) Overall structures of the Ragulator ($p18^{49\text{-}161}$) and Ragulator ($p18^{76\text{-}145}$) complexes. (**b**) Superposition of the Ragulator ($p18^{49\text{-}161}$) and Ragulator ($p18^{76\text{-}145}$) structures, which are colored in purple and gray, respectively, yielding an RMSD of 1.2 Å for 478 Cα atoms.

a

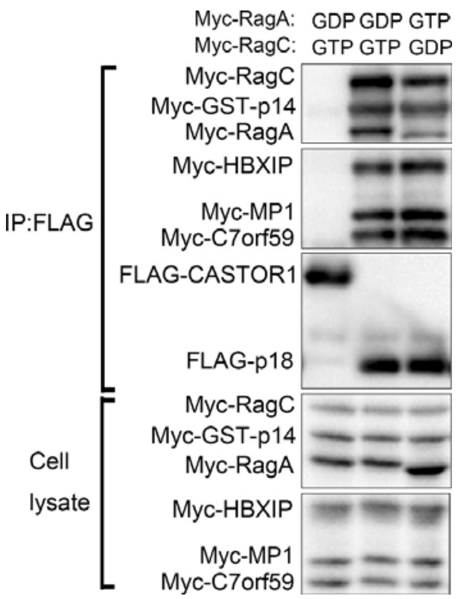

b

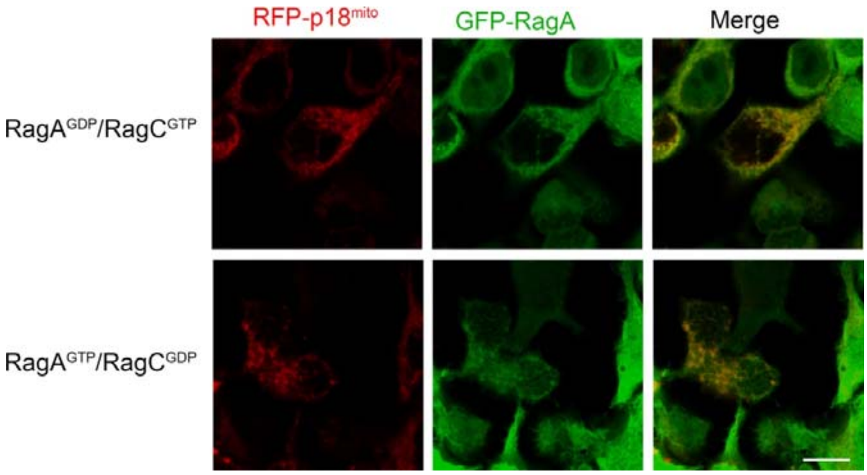

Supplementary Figure 10. The Rag GTPases interact with the Ragulator in a nucleotide-bound state independent manner. Co-IP assays (**a**) and immunofluorescence microscopy analyses (**b**) were performed to analyze the interactions between the Ragulator and the dominant active $RagA^{GTP}$-$RagC^{GDP}$ or dominant inactive $RagA^{GDP}$-$RagC^{GTP}$. Scale bar represents 10 μm.

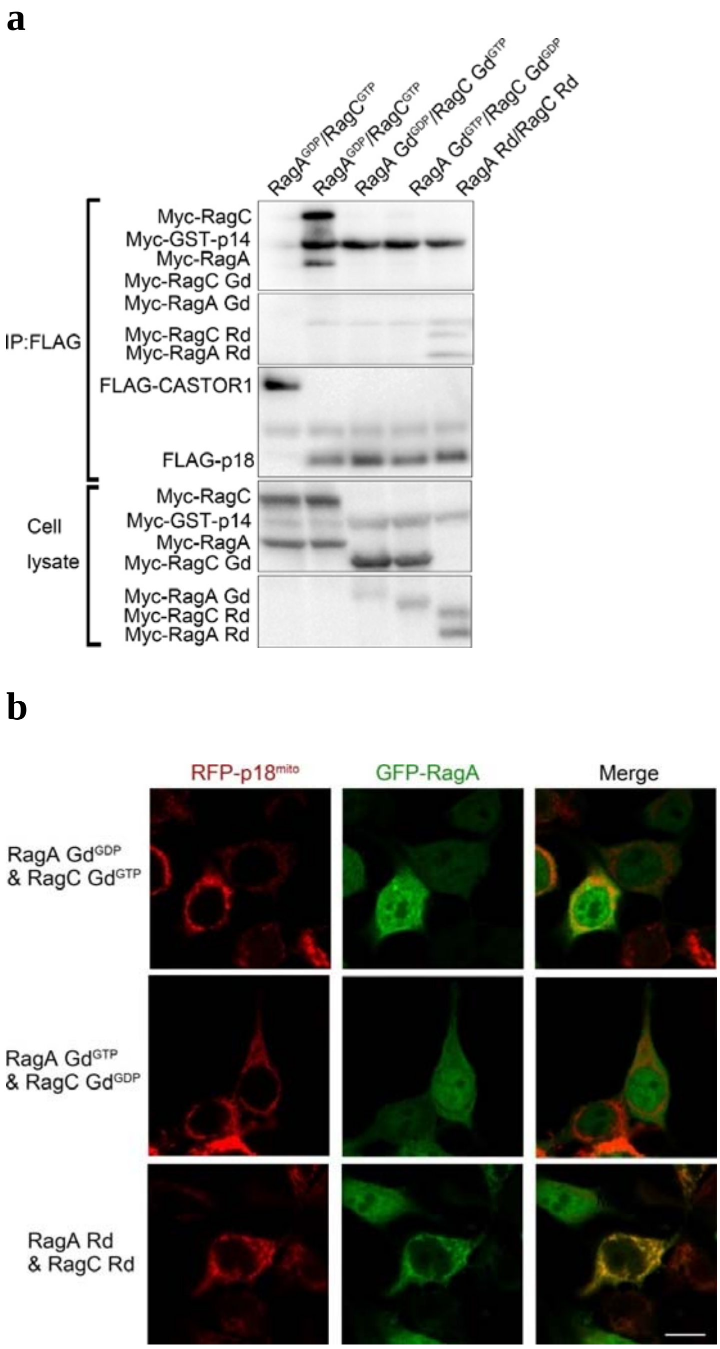

Supplementary Figure 11. The Rag GTPases interact with the Ragulator via the C-terminal Roadblock domains. Co-IP assays (**a**) and immunofluorescence microscopy analyses (**b**) were performed to analyze the interactions between the Ragulator and the Roadblock domains (Rd) or GTPase domains (Gd) of Rag GTPases. Scale bar represents 10 μm.

a

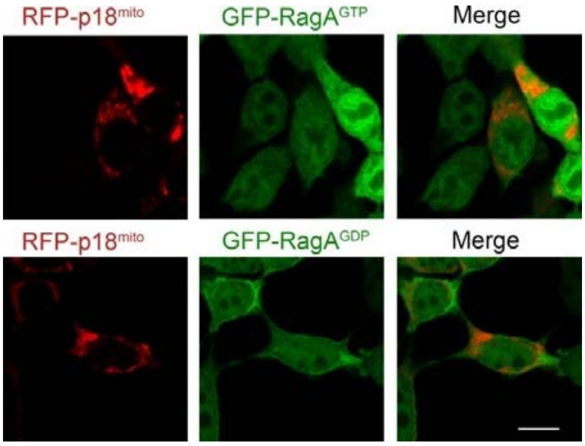
RFP-p18mito
GFP-RagAGTP
Merge
RFP-p18mito
GFP-RagAGDP
Merge

b

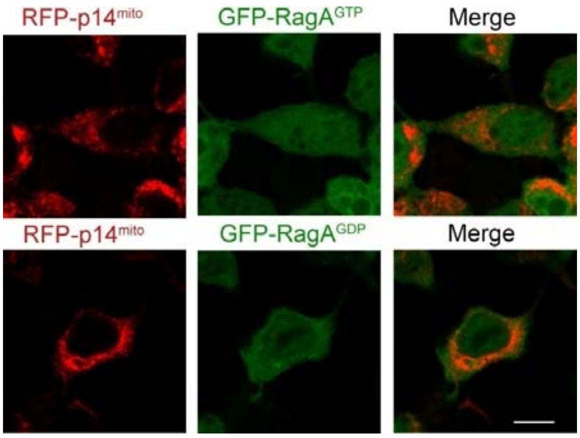
RFP-p14mito
GFP-RagAGTP
Merge
RFP-p14mito
GFP-RagAGDP
Merge

c

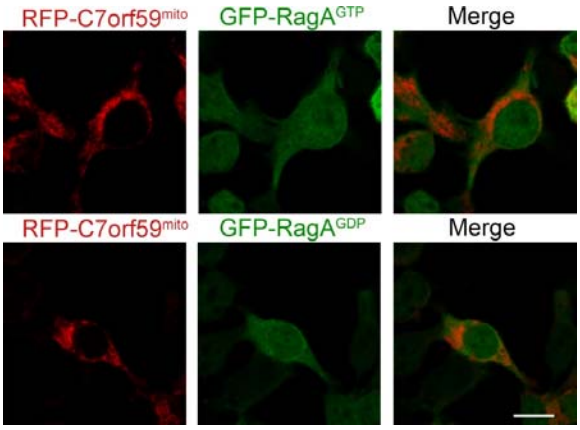
RFP-C7orf59mito
GFP-RagAGTP
Merge
RFP-C7orf59mito
GFP-RagAGDP
Merge

Supplementary Figure 12. Immunofluorescence microscopy analyses of the interactions of $p18^{mito}$, $p14^{mito}$, and $C7orf59^{mito}$ with the Rag GTPases. Scale bar represents 10 μm. (**a**) p18 alone failed to recruit $RagA^{GTP}$ or $RagA^{GDP}$ in cells over-expressing $p18^{mito}$, $RagA^{GTP}$-$RagC^{GDP}$ or $RagA^{GDP}$-$RagC^{GTP}$. (**b**) MP1-p14 alone failed to recruit $RagA^{GTP}$ or $RagA^{GDP}$ in cells over-expressing MP1, $p14^{mito}$, $RagA^{GTP}$-$RagC^{GDP}$ or $RagA^{GDP}$-$RagC^{GTP}$. (**c**) HBXIP-C7orf59 alone failed to recruit $RagA^{GTP}$ or $RagA^{GDP}$ in cells over-expressing HBXIP, $C7orf59^{mito}$, $RagA^{GTP}$-$RagC^{GDP}$ or $RagA^{GDP}$-$RagC^{GTP}$.

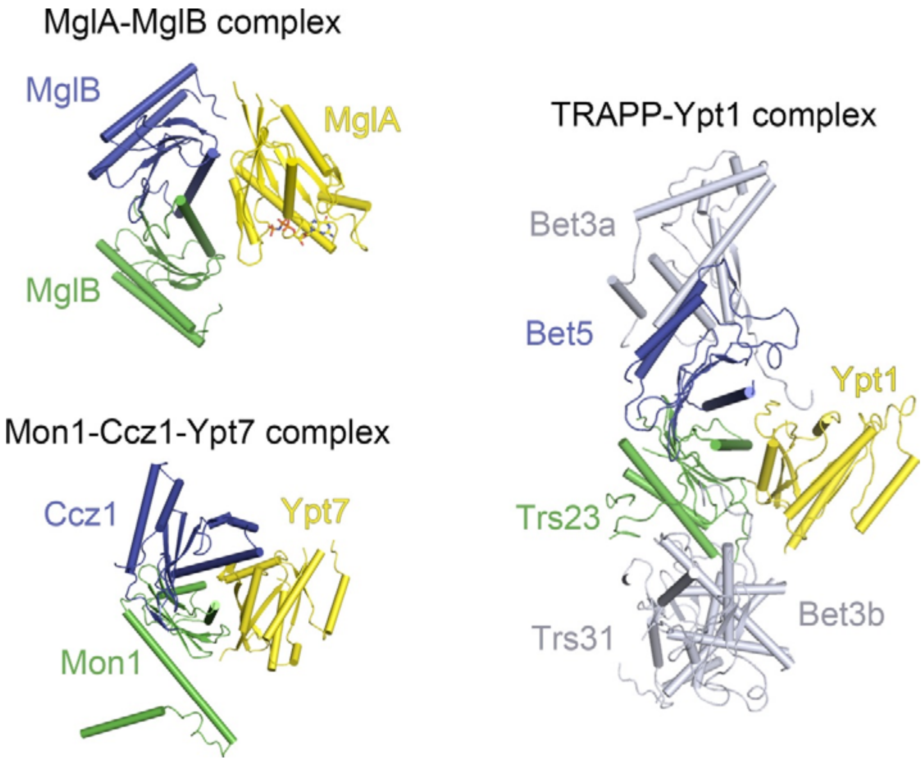

Supplementary Figure 13. Ribbon representations of three Roadblock or Longin domain-containing complexes, namely the MglA-MglB complex (PDB code 3T12), the Mon1-Ccz1-Ypt7 complex (PDB code 5LDD), and the TRAPP-Ypt1p complex (PDB code 3CUE).

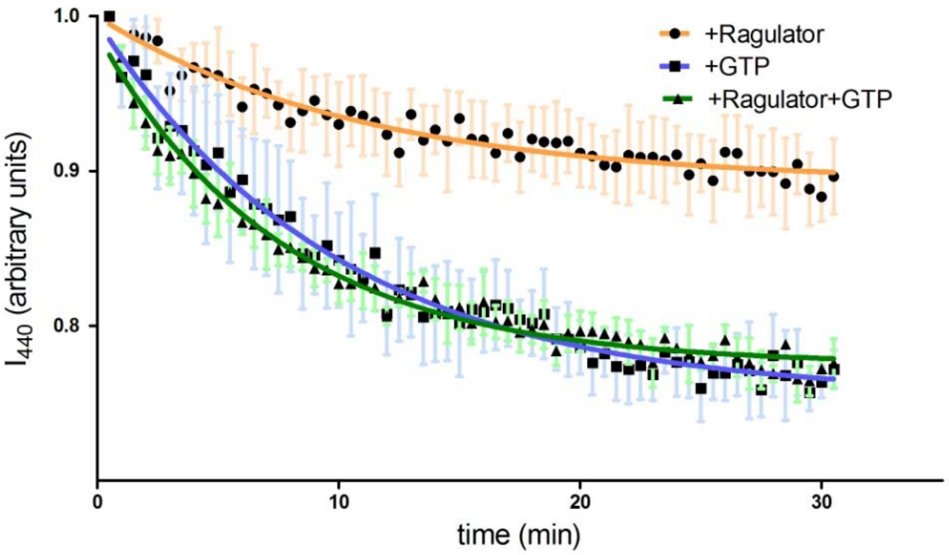

Supplementary Figure 14. Kinetics of mantGDP release from mantGDP-bound RagA-RagC^{D181N} in the presence of 2.0 μM Ragulator (yellow), or 10.0 μM GTP (blue), or 2.0 μM Ragulator and 10.0 μM GTP (green)**.** Each value represents the normalized mean ± deviation of three independent measurements.

a

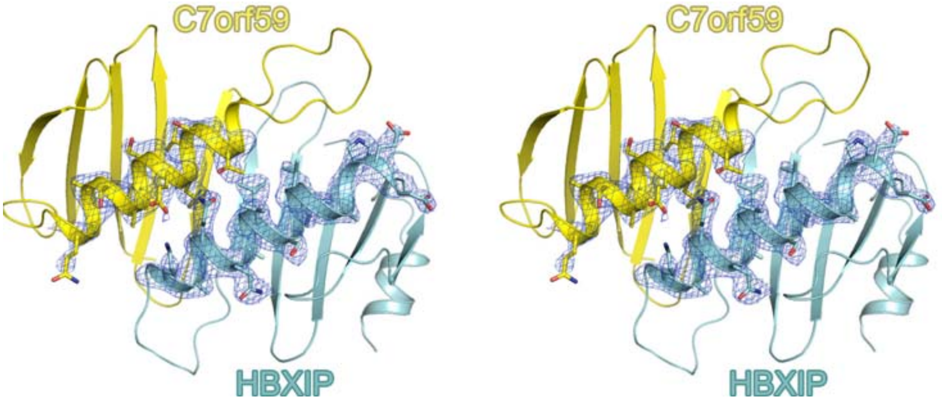
C7orf59
HBXIP
C7orf59
HBXIP

b

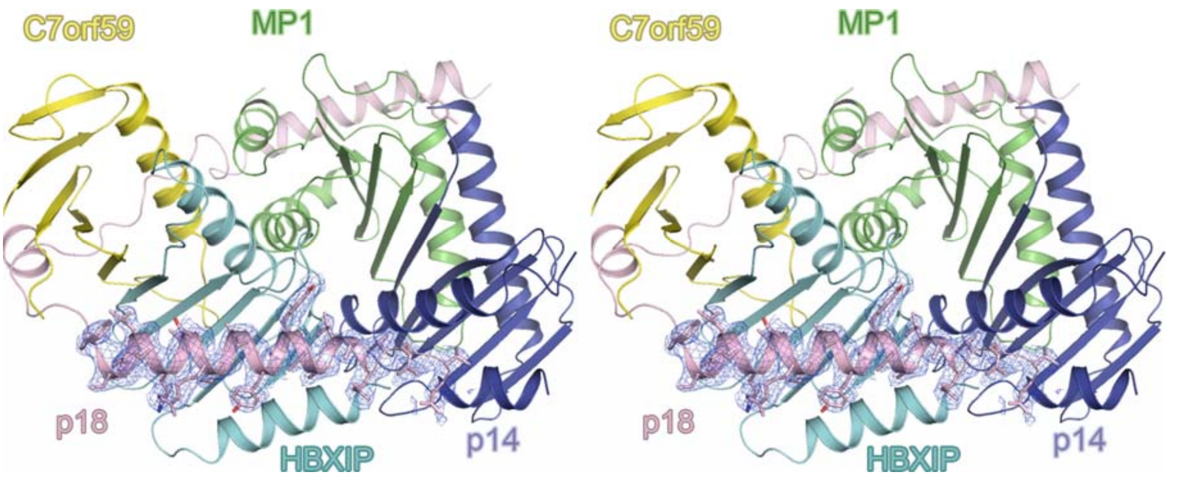
C7orf59
MP1
p18
HBXIP
p14
C7orf59
MP1
p18
HBXIP
p14

c

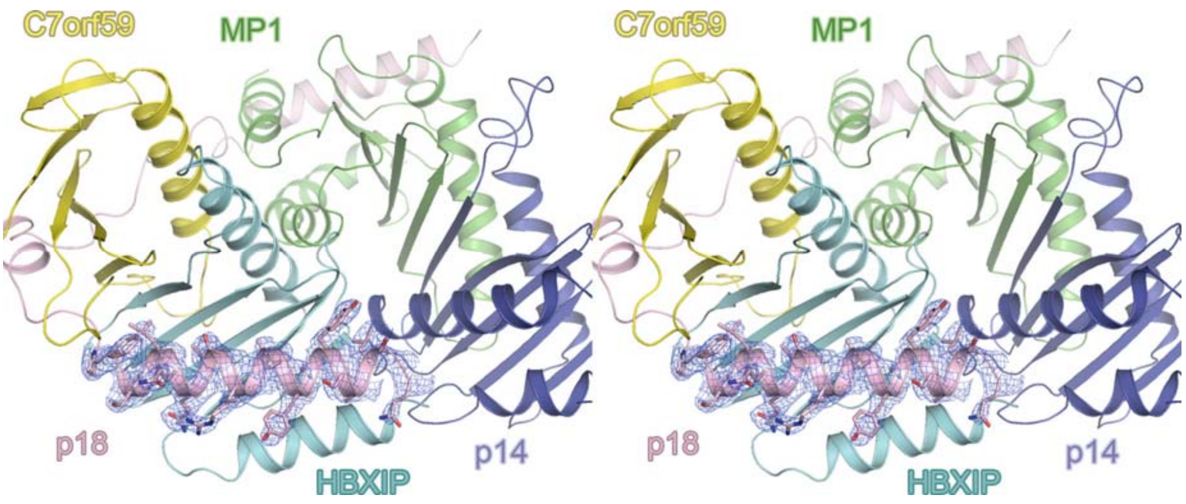
C7orf59
MP1
p18
HBXIP
p14
C7orf59
MP1
p18
HBXIP
p14

Supplementary Figure 15. Stereoviews of electron density maps. (**a**) Composite 2Fo-Fc omit map (contoured at 1σ) for the α2 helices of the HBXIP-C7orf59 complex. (**b**) Composite 2Fo-Fc omit map (contoured at 1σ) for helix α4 and the C-terminal loop of p18 in the Ragulator complex (p18$^{49-161}$). (**c**) Composite 2Fo-Fc omit map (contoured at 1σ) for helix α4 of p18 in the Ragulator complex (p18$^{49-145}$).

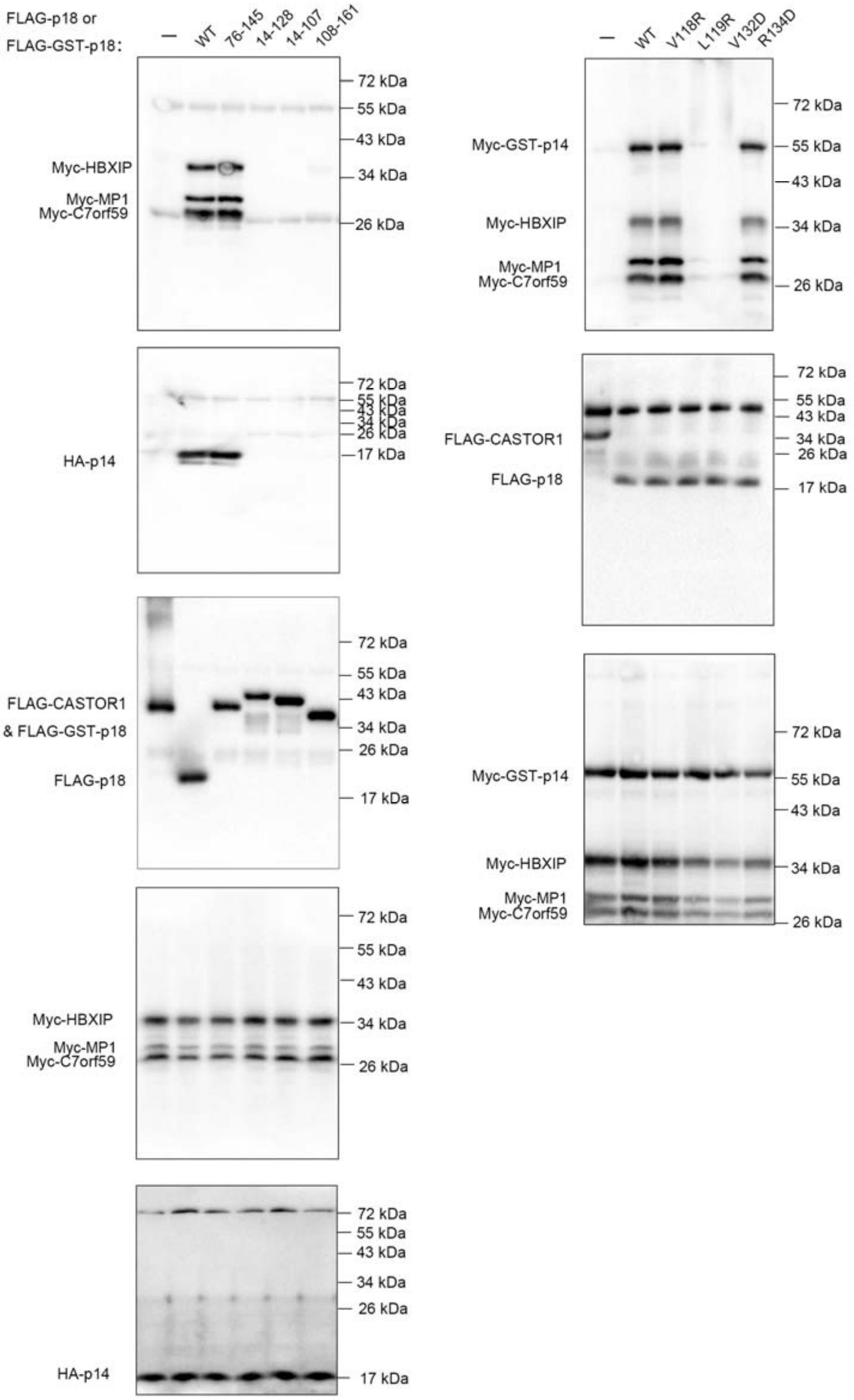

Full-length immunoblots for Figure 3a and 3b

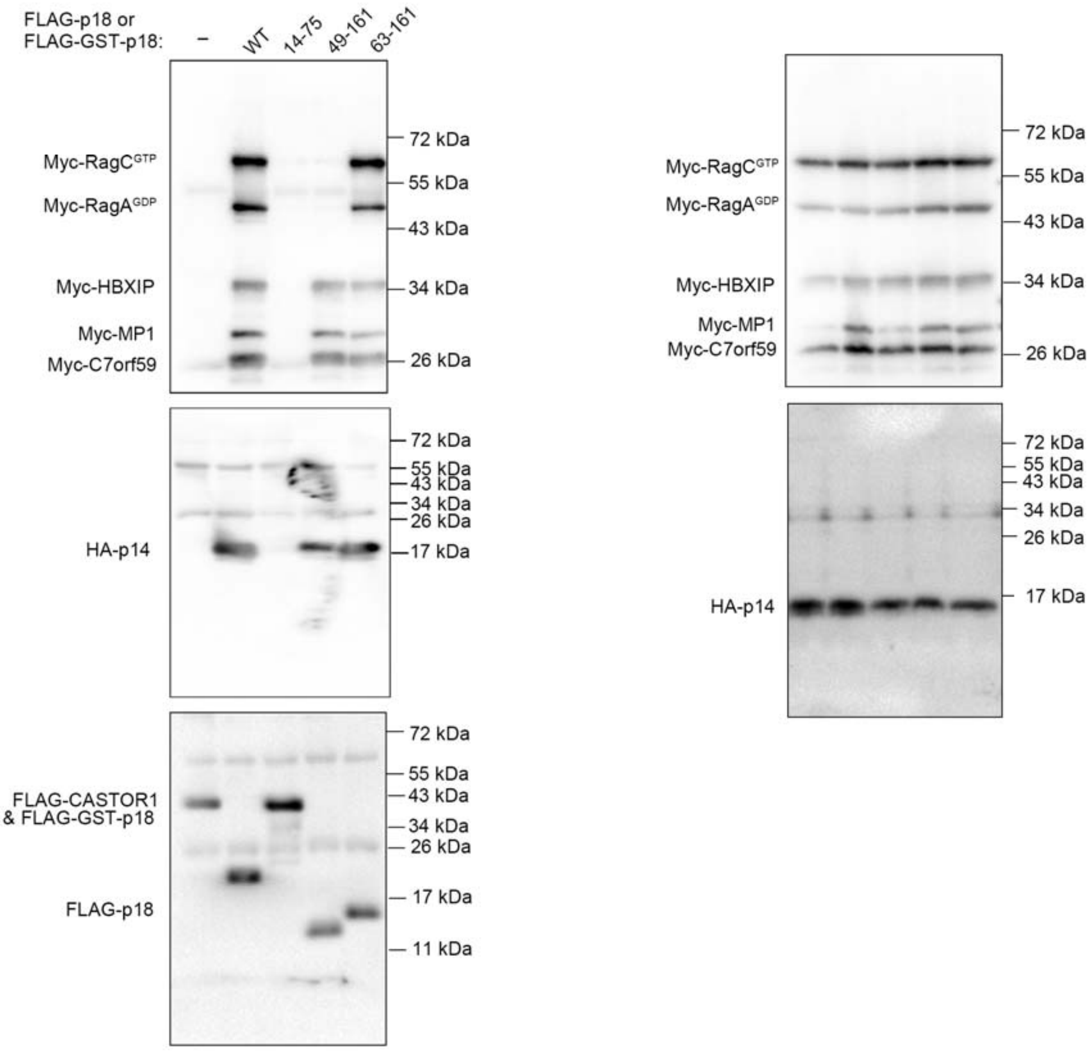

Full-length immunoblots for Figure 4a

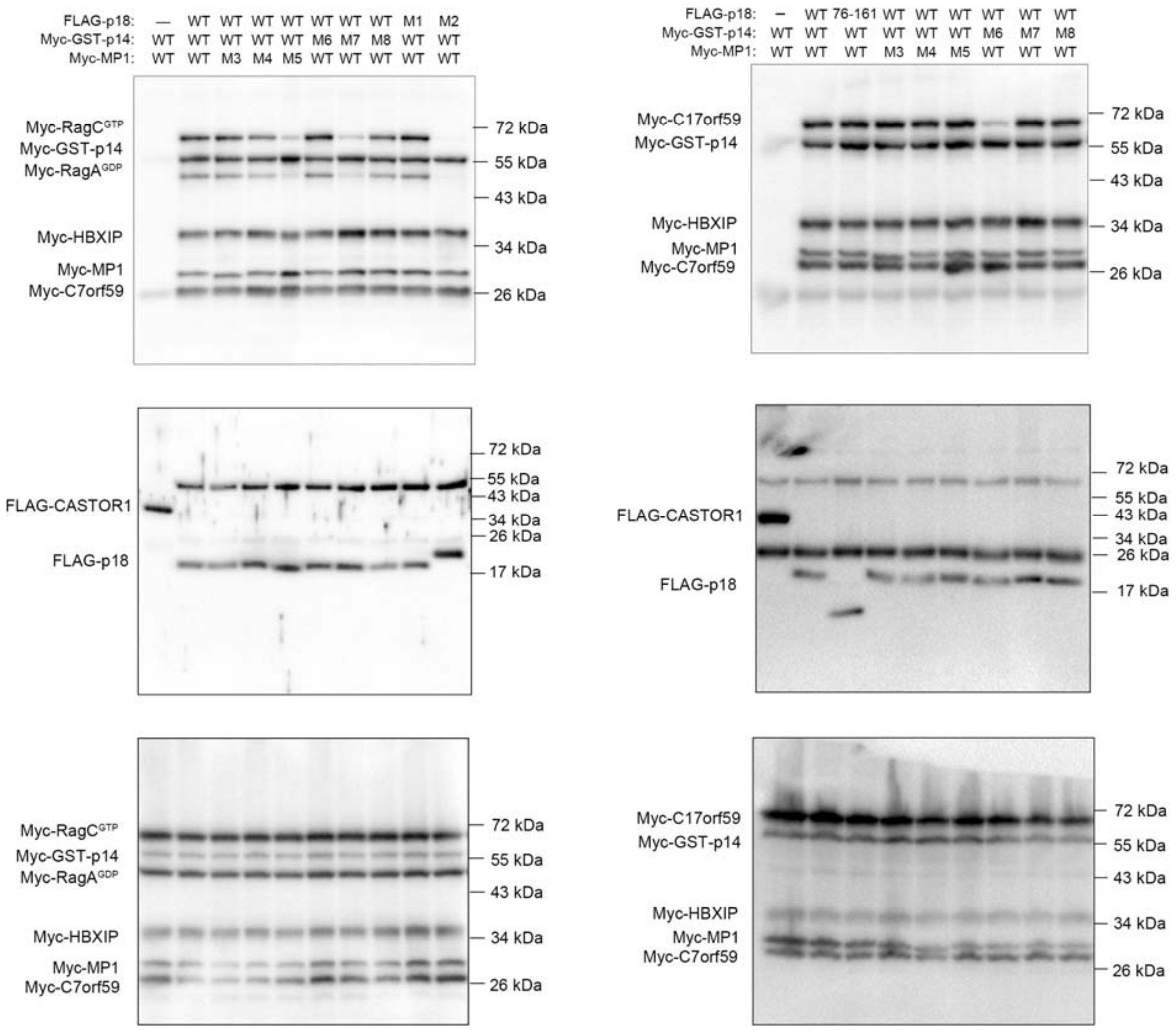

Full-length immunoblots for Figure 4c and 4e

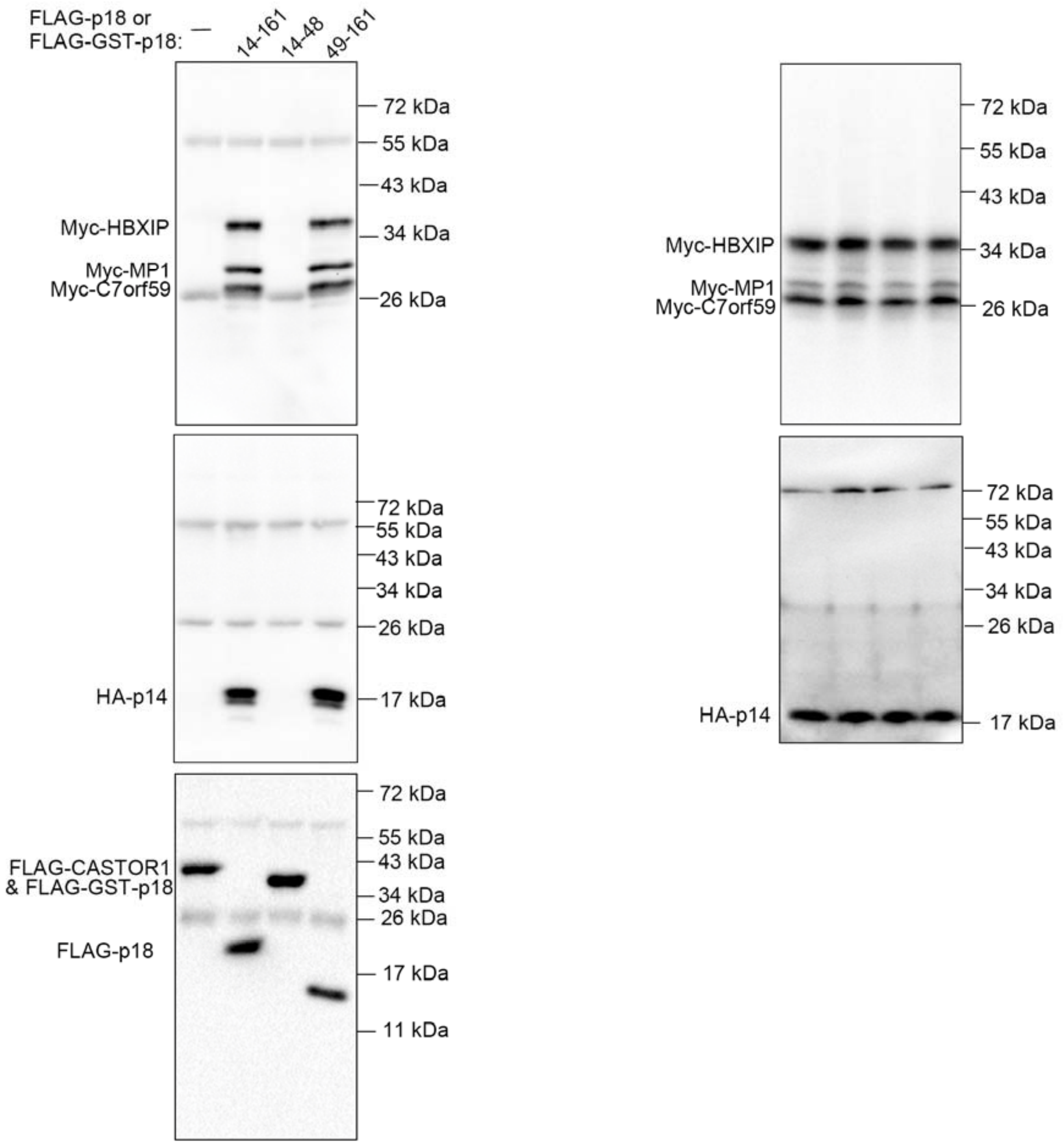

Full-length immunoblots for Supplementary Figure 2b

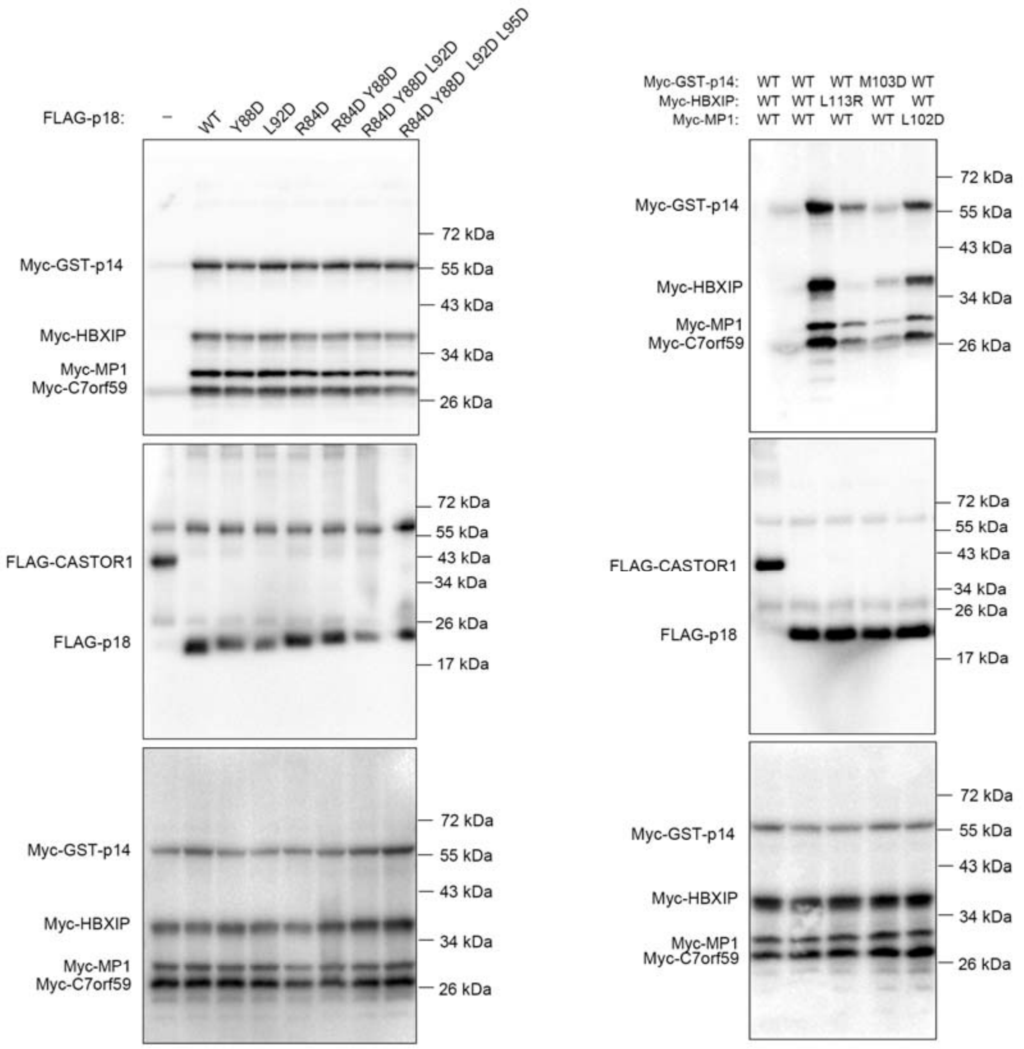

Full-length immunoblots for Supplementary Figure 7a and 7b

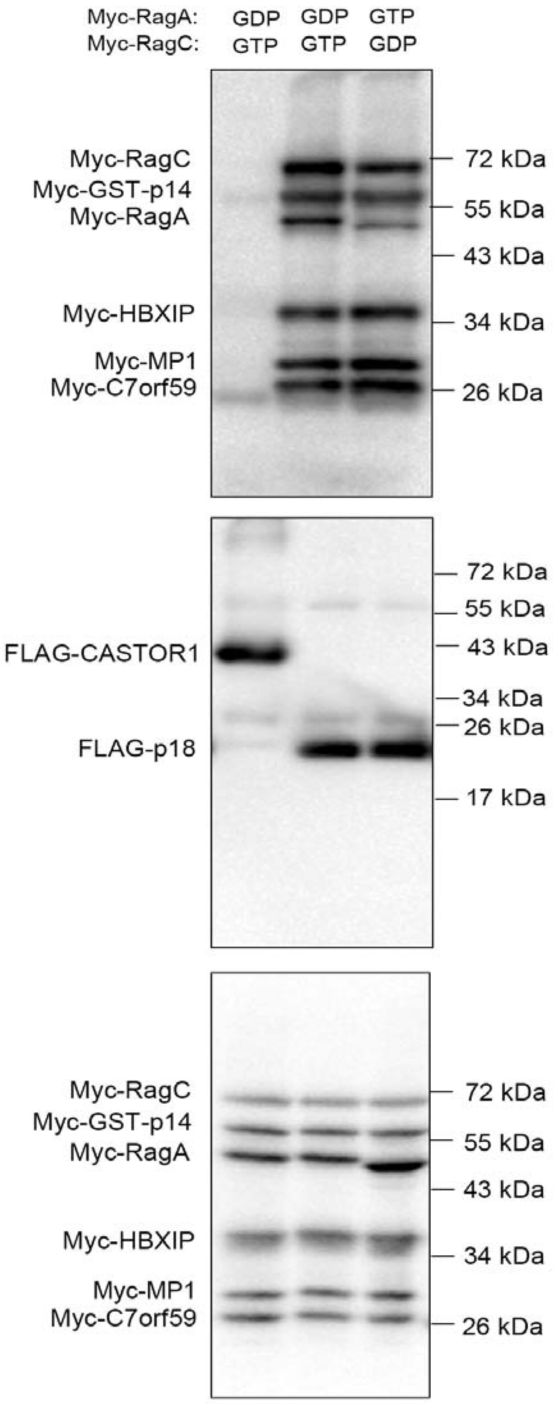

Full-length immunoblots for Supplementary Figure 10a

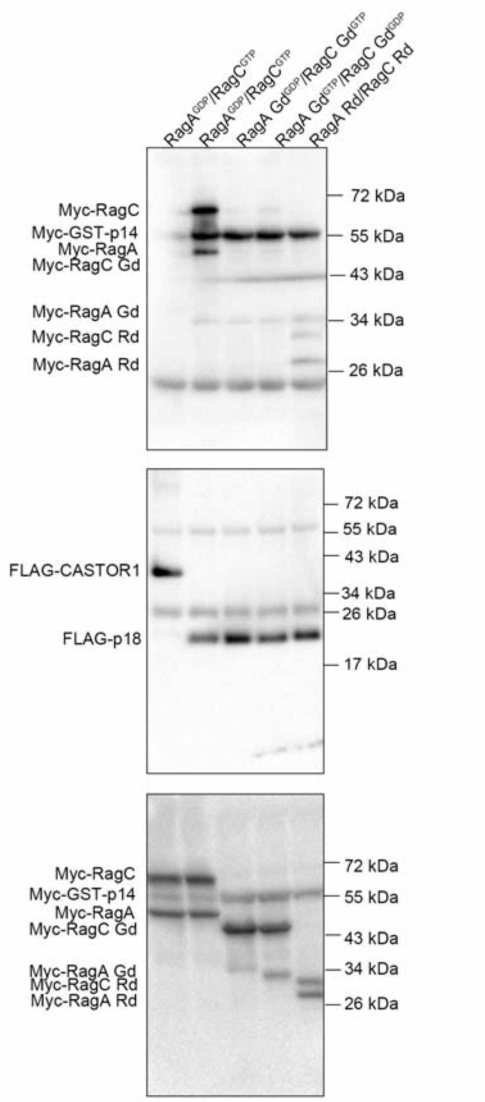

Full-length immunoblots for Supplementary Figure 11a

Supplemental Figure 16. Uncropped western blot images.

Supplementary Table 1. Design of mutants

Mutant	Mutation	Location
M1	D49A/E50A	α1 of p18
M2	I57D/L58D	α1 of p18
M3	E42A/H43A/R46A	β2-α2 loop and α2 of MP1
M4	L55D/D58A	α2 of MP1
M5	Q59A/K62D	α2 of MP1
M6	D41A/R43A	α2 of p14
M7	V44D/A47D	α2 of p14
M8	R58A/N59A/N61A/Q62A	α2 of p14

Supplementary Table 2. Summary of primers used in this study

gene	vector	primer sequence	
MP1	pET-Duet-1	Forward (5'-3')	ATATCCATGGCGGATGACCTAAAGC
		Reverse (5'-3')	ATATAAGCTTTTAAGAAACTTCCACAACTTGTCTC
p14	pET-Duet-1	Forward (5'-3')	ATATCATATGCTGCGCCCCAAG
		Reverse (5'-3')	TATACTCGAGTTAAGATGCCGCCACTTG
p18$^{14-161}$	pET-28a-SUMO	Forward (5'-3')	ATATGGATCCCAGGACCGAGAGGAGCG
		Reverse (5'-3')	ATATCTCGAGTCATGGGATCCCAAACTGTAC
p18$^{49-161}$	pET-28a-SUMO	Forward (5'-3')	ATATGGATCCGATGAGCAGGCCCTGCTCTC
		Reverse (5'-3')	ATATCTCGAGTCATGGGATCCCAAACTGTAC
p18$^{76-145}$	pET-28a-SUMO	Forward (5'-3')	TATAGGATCCATGGAGCAGCATGAGTACATG
		Reverse (5'-3')	ATATCTCGAGTCACTGAGAAAGTGCACTGTAGGCA
C7orf59	pET-28a	Forward (5'-3')	ATATCCATGGGAATGACTTCTGCGCTGA
		Reverse (5'-3')	ATATCTCGAGTCAGACATCAATGGGCTCC
HBXIP$^{83-173}$	pET-Duet-1	Forward (5'-3')	ATATCCATGGAGGCGACCTTGGAGC
		Reverse (5'-3')	ATATCTCGAGTCAAGAGGCCATTTTGTGCAC
RagC	pET-Duet-1	Forward (5'-3')	ATATCCATGGTCCCTGCAGTACGGG
		Reverse (5'-3')	ATATAAGCTTCTAGATGGCGTTTCGTGGC
RagA	pET-Duet-1	Forward (5'-3')	ATATCATATGCCAAATACAGCCATGAAGAA
		Reverse (5'-3')	TATACTCGAGTCAACGCATAAGGAGACTGTGC
p18$^{14-161}$	pcDNA-FLAG	Forward (5'-3')	CGCGGATCCTGCAGGACCGAGAGGAGCGG
		Reverse (5'-3')	CCGCTCGAGTCATGGTATCCCAAACTGTAC
p18 V118R	pcDNA-FLAG	Forward (5'-3')	CAGCCAGCCCCACCAACGGCTGGCCAGTGAGCCC
		Reverse (5'-3')	GGGCTCACTGGCCAGCCGTTGGTGGGGCTGGCTG
p18 L119R	pcDNA-FLAG	Forward (5'-3')	CCAGCCCCACCAAGTGCGGGCCAGTGAGCCCATC
p18 L119R-Mito	pmRFP-C1	Reverse (5'-3')	GATGGGCTCACTGGCCCGCACTTGGTGGGGCTGG
p18 V132D	pcDNA-FLAG	Forward (5'-3')	CTCTGATTTGCAGCAGGACTCCAGGATAGCTGC
		Reverse (5'-3')	GCAGCTATCCTGGAGTCCTGCTGCAAATCAGAG
p18 R134D	pcDNA-FLAG	Forward (5'-3')	GATTTGCAGCAGGTCTCCGACATAGCTGCTTATGC
		Reverse (5'-3')	GCATAAGCAGCTATGTCGGAGACCTGCTGCAAATC

p18 R84D	pcDNA-FLAG	Forward (5'-3')	CATGAGTACATGGACGATGCCAGGCAGTACAGC
		Reverse (5'-3')	GCTGTACTGCCTGGCATCGTCCATGTACTCATG
p18 Y88D	pcDNA-FLAG	Forward (5'-3')	GGACCGTGCCAGGCAGGACAGCACCCGCTTGGC
		Reverse (5'-3')	GCCAAGCGGGTGCTGTCCTGCCTGGCACGGTCC
p18 L92D	pcDNA-FLAG	Forward (5'-3')	GCAGTACAGCACCCGCGACGCTGTGCTGAGCAG
		Reverse (5'-3')	CTGCTCAGCACAGCGTCGCGGGTGCTGTACTGC
p18 R84D/Y88D	pcDNA-FLAG	Forward (5'-3')	CATGAGTACATGGACGATGCCAGGCAGGACAGC
		Reverse (5'-3')	GCTGTCCTGCCTGGCATCGTCCATGTACTCATG
p18 R84D/Y88D/L92D	pcDNA-FLAG	Forward (5'-3')	GCAGGACAGCACCCGCGACGCTGTGCTGAGCAG
		Reverse (5'-3')	CTGCTCAGCACAGCGTCGCGGGTGCTGTCCTGC
p18 R84D/Y88D/L92D/L95D	pcDNA-FLAG	Forward (5'-3')	CACCCGCGACGCTGTGGATAGCAGCAGCCTGACCC
		Reverse (5'-3')	GGGTCAGGCTGCTGCTATCCACAGCGTCGCGGGTG
p18 D49A/E50A	pcDNA-FLAG	Forward (5'-3')	CTTCCGCTCGCACTGCTGCGCAGGCCCTGCTCTC
		Reverse (5'-3')	GAGAGCAGGGCCTGCGCAGCAGTGCGAGCGGAAG
p18 I57D/L58D	pcDNA-FLAG	Forward (5'-3')	GCCCTGCTCTCTTCCGACGATGCCAAGACAGCCAGC
p18 I57D/L58D-Mito	pmRFP-C1	Reverse (5'-3')	GCTGGCTGTCTTGGCATCGTCGGAAGAGAGCAGGGC
$p18^{49-161}$	pcDNA-FLAG	Forward (5'-3')	CGCGGATCCTGGATGAGCAGGCCCTGCTCTC
		Reverse (5'-3')	CCGCTCGAGTCATGGTATCCCAAACTGTAC
GST-$p18^{14-48}$	pcDNA-FLAG	Forward (5'-3')	CGCGGATCCTGAACACTAGTAGCAATTCCATGTCC
		Reverse (5'-3')	CCGCTCGAGTTAAGTGCGAGCGGAAGGCAG
GST-$p18^{76-145}$	pcDNA-FLAG	Forward (5'-3')	CGCGGATCCTGAACACTAGTAGCAATTCCATGTCC
		Reverse (5'-3')	CCGCTCGAGTTACTGAGAAAGTGCACTGTAG
GST-$p18^{14-128}$	pcDNA-FLAG	Forward (5'-3')	CGCGGATCCTGAACACTAGTAGCAATTCCATGTCC
		Reverse (5'-3')	CCGCTCGAGTTAATCAGAGAACGGGATGGG
GST-$p18^{14-107}$	pcDNA-FLAG	Forward (5'-3')	CGCGGATCCTGAACACTAGTAGCAATTCCATGTCC
		Reverse (5'-3')	CCGCTCGAGCTACGGTGGCAGCTTCTTCC
GST-$p18^{108-161}$	pcDNA-FLAG	Forward (5'-3')	CGCGGATCCTGAACACTAGTAGCAATTCCATGTCC
		Reverse (5'-3')	CCGCTCGAGTCATGGTATCCCAAACTGTAC
GST-$p18^{108-145}$	pcDNA-FLAG	Forward (5'-3')	CGCGGATCCTGAACACTAGTAGCAATTCCATGTCC
		Reverse (5'-3')	CCGCTCGAGTTACTGAGAAAGTGCACTGTAG

p18$^{63-161}$	pcDNA-FLAG	Forward (5'-3')	CGCGGATCCTGATGGCCAGCAACATCATTGATG
		Reverse (5'-3')	CCGCTCGAGTCATGGTATCCCAAACTGTAC
GST-p18$^{14-75}$	pcDNA-FLAG	Forward (5'-3')	CGCGGATCCTGAACACTAGTAGCAATTCCATGTCC
		Reverse (5'-3')	CCGCTCGAGCTAGCCCTGTGAGTCTGCAGC
Mito	pmRFP-C1	Forward (5'-3')	CCGCTCGAGCTATGCATCGAGGCGACGGAG
		Reverse (5'-3')	CGCGGATCCTCAGAGCTGCTTTCGGTATCTCACG
p18$^{14-161}$-Mito	pmRFP-C1	Forward (5'-3')	CCGCTCGAGCTCAGGACCGAGAGGAGCGG
		Reverse (5'-3')	CGCGGATCCTCAGAGCTGCTTTCGGTATCTCACG
GST-p18$^{108-161}$-Mito	pmRFP-C1	Forward (5'-3')	CCGCTCGAGCTAACACTAGTAGCAATTCCATG
		Reverse (5'-3')	CGCGGATCCTCAGAGCTGCTTTCGGTATCTCACG
GST-p18$^{14-128}$-Mito	pmRFP-C1	Forward (5'-3')	CCGCTCGAGCTAACACTAGTAGCAATTCCATG
		Reverse (5'-3')	CGCGGATCCTCAGAGCTGCTTTCGGTATCTCACG
GST-p18$^{76-145}$-Mito	pmRFP-C1	Forward (5'-3')	CCGCTCGAGCTAACACTAGTAGCAATTCCATG
		Reverse (5'-3')	CGCGGATCCTCAGAGCTGCTTTCGGTATCTCACG
CASTOR1	pcDNA-FLAG	Forward (5'-3')	CGCGGATCCTGATGGAGCTGCACATCCTAGAAC
		Reverse (5'-3')	CCGCTCGAGTCAGGAAGCCAGGCCTTC
MP1	pcDNA-Myc	Forward (5'-3')	ATAAGAATGCGGCCGCATGGCGGATGACCTAAAGC
		Reverse (5'-3')	CCGCTCGAGTTAAGAAACTTCCACAACTTGTC
MP1 L102D	pcDNA-Myc	Forward (5'-3')	GTGCCAATACAGGAGATATTGTCAGCCTAGAAAAG
		Reverse (5'-3')	CTTTTCTAGGCTGACAATATCTCCTGTATTGGCAC
MP1 E42A/H43A/R46A	pcDNA-Myc	Forward (5'-3')	CAAATGACAATGCTCCAGCGGCTGCTTTGGCACCTGGTTTCTT
		Reverse (5'-3')	AAGAAACCAGGTGCCAAAGCAGCCGCTGGAGCATTGTCATTTG
MP1 L55D/D58A	pcDNA-Myc	Forward (5'-3')	CCACTTTTGCCGATGCAACAGCCCAAGGAAGCAAAC
		Reverse (5'-3')	GTTTGCTTCCTTGGGCTGTTGCATCGGCAAAAGTGG
MP1 Q59A/K62D	pcDNA-Myc	Forward (5'-3')	CAACAGACGCAGGAAGCGATCTTGGACTTTCC
		Reverse (5'-3')	GGAAAGTCCAAGATCGCTTCCTGCGTCTGTTG
MP1	pcDNA-HA	Forward (5'-3')	GATACCCCTACGACGTCCCCGACTACGCCATGGCGGATGACCTAAAGC
		Reverse (5'-3')	CCGCTCGAGTTAAGAAACTTCCACAACTTGTC
MP1	pEGFP-N3	Forward (5'-3')	CCGCTCGAGCTATGGCGGATGACCTAAAGC
		Reverse (5'-3')	CGCGGATCCAGAAACTTCCACAACTTGTCTC

GST-p14	pcDNA-Myc	Forward (5'-3')	CCGGAATTCTAACACTAGTAGCAATTCCATGTCC
		Reverse (5'-3')	CCGCTCGAGTTAAGATGCCGCCACTTGGG
p14 M103D	pcDNA-Myc	Forward (5'-3')	GACCGTGGGCTTTGGAGACCTCAAGGCCAAGGC
		Reverse (5'-3')	GCCTTGGCCTTGAGGTCTCCAAAGCCCACGGTC
p14 D41A/R43A	pcDNA-Myc	Forward (5'-3')	GTTACGGGGACACTGCCGCCGCGGTCACCGCTGCCATAG
		Reverse (5'-3')	CTATGGCAGCGGTGACCGCGGCGGCAGTGTCCCCGTAAC
p14 V44D/A47D	pcDNA-Myc	Forward (5'-3')	CTGACGCCCGGGACACCGCTGACATAGCCAGTAACATC
GST-p14 V44D/A47D		Reverse (5'-3')	GATGTTACTGGCTATGTCAGCGGTGTCCCGGGCGTCAG
p14 R58A/N59A/N61A/Q62A	pcDNA-Myc	Forward (5'-3')	GGCCGCCTACGACGCGGCCGGGGCCGCAGCGTTTAATG
		Reverse (5'-3')	CATTAAACGCTGCGGCCCCGGCCGCGTCGTAGGCGGCC
p14	pcDNA-Myc	Forward (5'-3')	ATAAGAATGCGGCCGCATGCTGCGCCCCAAGGCTTTG
		Reverse (5'-3')	CCGCTCGAGTTAAGATGCCGCCACTTGGG
p14	pcDNA-HA	Forward (5'-3')	GATACCCCTACGACGTCCCCGACTACGCCATGCTGCGCCCCAAGGC
		Reverse (5'-3')	CCGCTCGAGTTAAGATGCCGCCACTTGGG
p14-Mito	pmRFP-C1	Forward (5'-3')	CCGCTCGAGCTATGCTGCGCCCCAAGGC
		Reverse (5'-3')	CGCGGATCCTCAGAGCTGCTTTCGGTATCTCACG
HBXIP	pcDNA-Myc	Forward (5'-3')	CCGGAATTCTATGGAGCCAGGTGCAGGTC
		Reverse (5'-3')	CCGCTCGAGTCAAGAGGCCATTTTGTGCAC
HBXIP L113R	pcDNA-Myc	Forward (5'-3')	CACAAGGACTTAATCGGGGTTGCCGCGGGACCC
		Reverse (5'-3')	GGGTCCCGCGGCAACCCCGATTAAGTCCTTGTG
HBXIP	pcDNA-HA	Forward (5'-3')	CGCGGATCCATGGAGCCAGGTGCAGGTC
		Reverse (5'-3')	CCGCTCGAGTCAAGAGGCCATTTTGTGCAC
C7orf59	pcDNA-Myc	Forward (5'-3')	CCGGAATTCTATGACTTCTGCGCTGACCC
		Reverse (5'-3')	CCGCTCGAGTCAGACATCAATGGGCTCCC
C7orf59	pcDNA-FLAG	Forward (5'-3')	CGCGGATCCTGATGACTTCTGCGCTGACCC
		Reverse (5'-3')	CCGCTCGAGTCAGACATCAATGGGCTCCC
C7orf59	pcDNA-HA	Forward (5'-3')	CGCGGATCCATGACTTCTGCGCTGACCC
		Reverse (5'-3')	CCGCTCGAGTCAGACATCAATGGGCTCCC

C7orf59-Mito	pmRFP-C1	Forward (5'-3')	CCGCTCGAGCTATGACTTCTGCGCTGACCC
		Reverse (5'-3')	CGCGGATCCTCAGAGCTGCTTTCGGTATCTCACG
RagA	pcDNA-Myc	Forward (5'-3')	ATAAGAATGCGGCCGCATGCCAAATACAGCCATGAAG
		Reverse (5'-3')	CCGCTCGAGTCAACGCATAAGGAGACTGTGC
RagA T21N	pcDNA-Myc	Forward (5'-3')	GCGGGTCGGGGAAGAACAGCATGAGGTCGATAATC
	pEGFP-C3	Reverse (5'-3')	GATTATCGACCTCATGCTGTTCTTCCCCGACCCGC
RagA Q66L	pcDNA-Myc	Forward (5'-3')	GTGGGACTGTGGCGGTCTGGACACCTTCATGG
	pEGFP-C3	Reverse (5'-3')	CCATGAAGGTGTCCAGACCGCCACAGTCCCAC
RagA G domain	pcDNA-Myc	Forward (5'-3')	GTCTACCAGCTGATTTAGAACGTTCAGCAGCTGG
		Reverse (5'-3')	CCAGCTGCTGAACGTTCTAAATCAGCTGGTAGAC
RagA Roadblock domain	pcDNA-Myc	Forward (5'-3')	CATTTCTGAAGAGGACTTGAATTCTATGCCCAACGTTCAGCAGCTGGAG
		Reverse (5'-3')	GGGCCCTCTAGATGCATGCTCGAGTCAACGCATAAGGAGACTG
RagA	pEGFP-C3	Forward (5'-3')	CCGCTCGAGATGCCAAATACAGCCATGAAG
		Reverse (5'-3')	CGCGGATCCTCAACGCATAAGGAGACTGTGC
RagA G domain	pEGFP-C3	Forward (5'-3')	GTCTACCAGCTGATTTAGAACGTTCAGCAGCTGG
		Reverse (5'-3')	CCAGCTGCTGAACGTTCTAAATCAGCTGGTAGAC
RagA Roadblock domain	pEGFP-C3	Forward (5'-3')	CCGCTCGAGATGCCCAACGTTCAGCAGCTGGAG
		Reverse (5'-3')	CGCGGATCCTCAACGCATAAGGAGACTGTGC
RagC	pcDNA-Myc	Forward (5'-3')	ATAAGAATGCGGCCGCATGTCCCTGCAGTACGGGG
		Reverse (5'-3')	CCGCTCGAGCTAGATGGCGTTTCGTGGCG
RagC S75N	pcDNA-Myc	Forward (5'-3')	CGGCGCAGCGGCAAGAACTCCATCCAGAAGGTG
		Reverse (5'-3')	CACCTTCTGGATGGAGTTCTTGCCGCTGCGCCG
RagC Q120L	pcDNA-Myc	Forward (5'-3')	GATATGGGATTTTCCTGGGCTAATGGACTTTTTTG
		Reverse (5'-3')	CAAAAAAGTCCATTAGCCCAGGAAAATCCCATATC
RagC G domain	pcDNA-Myc	Forward (5'-3')	GGTGCAGAAACTCATTTGACAACTGCCGACCTTG
		Reverse (5'-3')	CAAGGTCGGCAGTTGTCAAATGAGTTTCTGCACC
RagC Roadblock domain	pcDNA-Myc	Forward (5'-3')	CCGGAATTCTATGCCACAACTGCCGACCTTGG
		Reverse (5'-3')	CCGCTCGAGCTAGATGGCGTTTCGTGGCG
C17orf59	pcDNA-Myc	Forward (5'-3')	CCGGAATTCTATGGAGTCGTCTCGGGGGC
		Reverse (5'-3')	CCGCTCGAGTCACTTGCACAGGGCCTCCAAC

Supplementary References

1. Gouet, P., Courcelle, E., Stuart, D.I. & Metoz, F. ESPript: analysis of multiple sequence alignments in PostScript. *Bioinformatics* **15**, 305-8 (1999).
2. Yachdav, G. et al. PredictProtein--an open resource for online prediction of protein structural and functional features. *Nucleic Acids Res.* **42**, W337-43 (2014).
3. Kurzbauer, R. et al. Crystal structure of the p14/MP1 scaffolding complex: How a twin couple attaches mitogen-activated protein kinase signaling to late endosomes. *Proc. Natl. Acad. Sci. USA* **101**, 10984-10989 (2004).
